# Predictive use of environmental regularities requires action relevance

**DOI:** 10.1038/s41598-026-35500-x

**Published:** 2026-01-13

**Authors:** Benedikt Kretzmeyer, Constantin A. Rothkopf, Katja Fiehler

**Affiliations:** 1 Experimental Psychology, Justus Liebig University Giessen, Otto-Behaghel-Str. 10F, 35394 Giessen, Germany; 2 Psychology of Information Processing, Technical University of Darmstadt, Alexanderstr. 10, 64283 Darmstadt, Germany

**Keywords:** Motor planning, Predictive control, Virtual reality, Embodied decision making, Adaptive behavior, Human statistical learning, Neuroscience, Psychology, Psychology

## Abstract

Efficient navigation often depends on the ability to learn and exploit environmental regularities through predictive motor adjustments. We conducted two virtual reality experiments in which participants walked through a museum corridor towards one of two exit doors. In Experiment 1, a moveable obstacle appeared with high probability on one side depending on the block, but participants could delay their pathway choice until the obstacle became visible. Many adopted a waiting strategy, walking straight until the obstacle appeared and only then adjusting their route. This raised the question of whether this reflected a failure to learn or a strategic delay. In Experiment 2, we introduced a larger static obstacle that forced participants to commit earlier, thereby increasing the cost of late corrections. Under these constraints, nearly all participants consistently selected the correct pathway in anticipation, indicating that the spatial structure could be learned and exploited when early commitment was required. Together, these findings suggest that predictive motor planning does not simply arise from the presence of environmental regularities but reflects an adaptive process of embodied decision-making shaped by task demands, environmental structure, individual strategy preferences, and learning capabilities.

**Supplementary Information:**

The online version contains supplementary material available at 10.1038/s41598-026-35500-x.

## Introduction

Imagine crossing a busy street when parked cars obstruct your view of oncoming traffic. You are unable to see from where cars are coming, but you begin to walk anyway, perhaps already angling your path slightly based on your familiarity with the local traffic patterns. In many situations like this, we do not wait for full information. Instead, we act based on prior experience, using probabilistic expectations to guide movement under uncertainty. Human behavior often unfolds in this way: we begin to move before all relevant information is available, using internal models of the world to bias our decisions towards likely or advantageous outcomes. This principle is well documented in research on embodied decision-making, which emphasizes that action and decision-making unfold in parallel rather than as distinct sequential stages^1^. A central question in this domain is how individuals use prior knowledge, especially probabilistic regularities, to guide motor planning when sensory evidence is incomplete.

While this question has been studied in visually guided reaching tasks, where participants adjust their movement trajectories based on probabilistic information about target likelihoods^2,3^, less is known about how these mechanisms operate during whole-body movement and spatial navigation. Yet similar principles appear to apply. In locomotor tasks, individuals adjust anticipatory postural plans based on potential action costs and the risk of destabilization under uncertainty^4^. In screen-based navigation tasks, individuals learn to adjust their behavior based on spatial outcome probabilities and cue reliability, flexibly adapting their choices as statistical regularities are internalized^5–7^. In naturalistic walking tasks, gaze allocation has also been shown to adapt flexibly to environmental statistics, with observers prioritizing attention to locations associated with increased risk based on learned probabilities^8^.

Still, much of this work relies on explicit cueing or conventional lab environments that constrain naturalistic movement and learning. Real-world behavior, in contrast, often requires learning regularities through experience alone, without instruction. Moreover, most studies focus on constrained motor tasks like reaching, whereas many everyday tasks, such as navigation, involve sequential and dynamic, full-body movement. To bridge this gap, we implemented a virtual reality (VR) navigation task in which participants repeatedly chose between two paths, one of which was blocked with varying probabilities across blocks. Crucially, participants were not told the underlying probabilities but had to infer them through repeated exposure. VR allowed us to preserve experimental control while studying learning and motor adjustments in an immersive, spatially structured setting that supports full-body movement^9,10^. We hypothesized that as participants learned the probabilistic structure of a block, they would begin to incorporate this knowledge into their movement plans, adjusting their movements earlier and more consistently towards the likely open path in later trials.

This hypothesis aligns with models of action selection in the dorsal sensorimotor stream, which propose that multiple potential movements are represented in parallel and compete for execution^11,12^. The outcome of this competition is biased by both expected value and spatial layout: the greater the angular distance between competing options, the more distinct their motor plans, and the more costly it becomes to switch late. In our task, learning the probabilistic structure of the environment may help bias motor planning early towards the more likely path, supporting smoother, more efficient trajectories and reducing the need for abrupt, energetically costly corrections. Early trajectory adjustments help avoid such corrections, which are not only cognitively demanding^13,14^ but also metabolically costly. Even small deviations from the body’s pendulum-like gait, characterized by rhythmic, energy-saving vertical oscillations of the center of mass, have been shown to increase energy expenditure^15^. Sharp turns are typically avoided altogether, as humans tend to follow smooth and efficient paths that preserve rhythmic flow, a strategy that is biomechanically optimal^16^.

Similar principles apply to gaze behavior. In naturalistic settings, gaze allocation adapts to learned environmental structure: observers prioritize attention towards likely outcomes and reduce exploration as uncertainty decreases^8,17^. We therefore predicted that visual search behavior would decrease as learning progresses, reflected in reduced saccade rate and fixation dispersion across trials within a block.

## Experiment 1

### Methods

#### Participants

Thirty participants were recruited for the first experiment. One dataset was excluded from analysis due to technical issues, resulting in a final sample of twenty-nine participants (21 female, 8 male) with a mean age of 24.28 years (*SD* = 3.39, range = 20–34 years). All participants had normal or corrected-to-normal vision and no movement restrictions. Based on the Edinburgh Handedness Inventory (EHI)^18^, 26 participants were right-handed. Written informed consent was obtained from all participants, who received either monetary compensation (€8/hour) or course credit. The study was approved by the local Ethics Review Board at the Department of Psychology and Sports Science, Justus Liebig University Giessen, Germany (Certificate # 2019-0003) and was conducted in accordance with the Declaration of Helsinki (2013), except for preregistration.

#### Experimental setup

Participants were immersed in virtual reality using a Vive Pro Eye head-mounted display (HMD; HTC Corporation, Taoyuan City, Taiwan), with a 90 Hz frame rate, 1440 × 1600 pixels per eye resolution, and a 110° field of view. The HMD features an integrated eye-tracking system with a 120 Hz sampling rate and a reported spatial accuracy of 0.5°–1.1°^19^. Four SteamVR 2.0 base stations (“lighthouses”; Valve Corporation, Bellevue, WA, USA) provided spatial tracking. Participants’ movements were captured using three Vive trackers attached to the waist, left foot, and right foot. They did not see a virtual version of themselves. Each participant held a Vive controller in their dominant hand. To allow for unrestricted movement without tripping hazards, the HMD cable was secured to the ceiling using a carabiner. The virtual environment was implemented in Unity (version 2021.3.12f1; Unity Technologies Inc., San Francisco, CA, USA), and the experiment utilized the SteamVR plugin (version 1.20.1) and the SRanipal SDK (version 1.3.1.1) for eye tracking integration. The block and trial structure, as well as data collection, were managed using the Unity Experiment Framework^20^. The experiment was run on a desktop PC (Intel Core i9-13900K CPU, 64 GB RAM, NVIDIA GeForce RTX 4090 GPU). The virtual room measured 4.9 m by 3.6 m, corresponding to the room’s physical dimensions. Participants moved physically within this space, and their real-world locomotion was mapped into the virtual environment via SteamVR tracking. The matched room dimensions allowed participants to walk naturally at their self-selected pace.

*Visual environment *Participants were immersed in a small virtual museum environment measuring 4.9 m in length, 3.6 m in width, and 3.5 m in height. The space featured a wooden brown floor texture and was illuminated by spotlights mounted on the ceiling. Several paintings were displayed along the left and right walls, while the front and back walls featured signs with the museum’s name (MoMVA—Museum of Modern Virtual Art) as well as fictional museum maps.

**Fig. 1:**
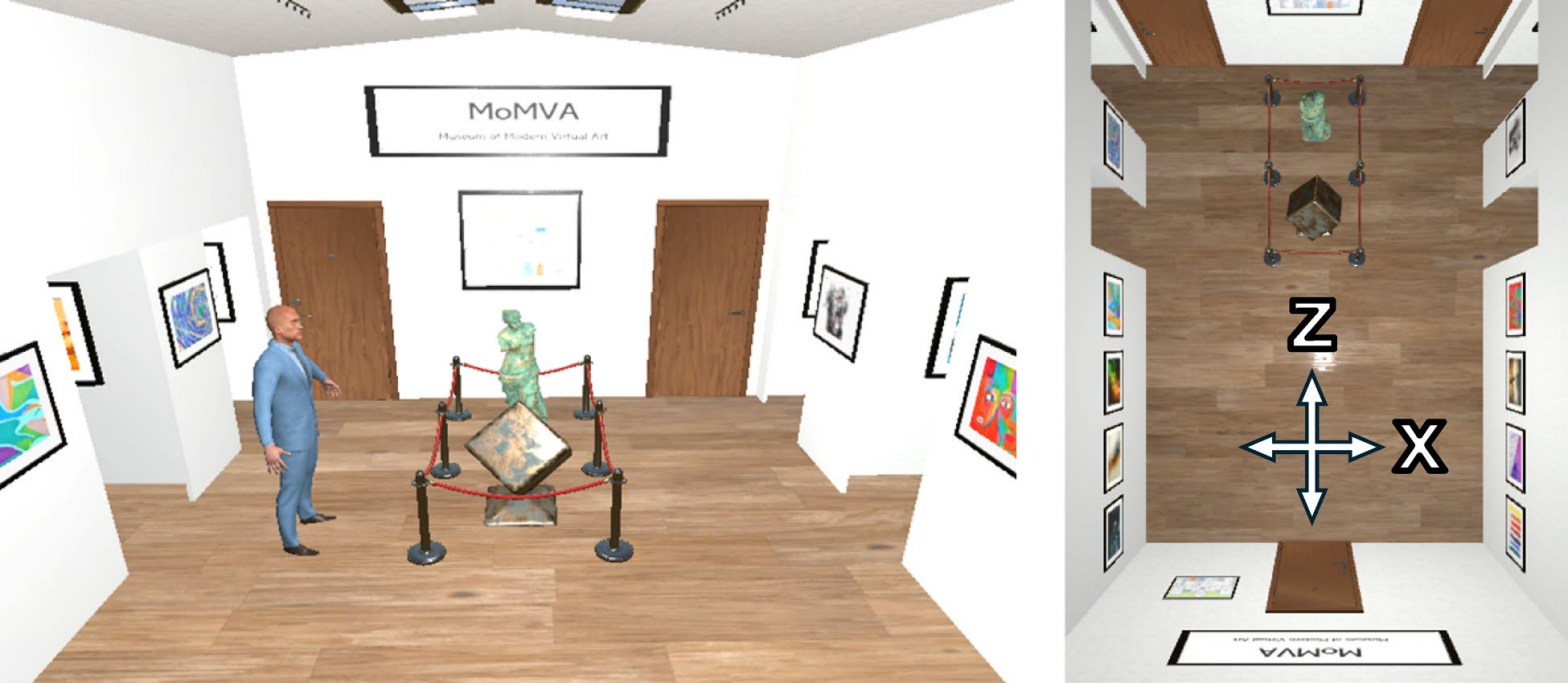
Depiction of the virtual museum. In the left image, the security guard emerged from one of the two left entry points and blocked the pathway towards the left exit door. The right image presents a top-down view of the museum layout prior to the guard’s appearance.

*Experimental objects *The virtual controller held by participants in their dominant hand visually matched the real-world Vive controller. The virtual environment is depicted in Fig. 1. A brown door positioned at the center of the back wall marked the starting point for each trial and was located directly behind the participant. On the front wall, two additional brown doors served as exit points, placed symmetrically to the left and right. Each exit door featured a doorknob positioned 1.5 m to the left or right of the participant’s starting location, respectively, ensuring equal distance to both exits. The straight-line distance from the participant’s starting point to either doorknob was approximately 4.93 m, assuming a direct diagonal path without lateral deviation. At a fixed distance of 2.7 m from the back wall, a central obstacle blocked the middle section of the museum. This obstacle, measuring 0.85 m in width, consisted of several statues enclosed by rope barriers supported by stanchions. Open doorways on the left and right walls served as entry points for a security guard. The guard, dressed in a blue suit, was animated using the ‘Man in a Suit’ model^21^ and the ‘Human Basic Motions FREE’ animation package^22^, specifically the ‘Idle’ animation.

#### Task

The participants’ task was to navigate from the starting position to either the left or right exit door and touch the doorknob with the VR controller while avoiding obstacles. The participants were instructed to take the shortest path possible. On each trial, the security guard moved into the room from either the left or right entry points and blocked one of the two pathways to the exit doors. The guard’s appearance was dynamically triggered based on the participant’s position in the virtual room, with the triggering position randomly sampled within a range of 0.8 to 1.2 m. This ensured that participants could not predict the time point of the guard’s appearance. Participants were instructed to avoid both the guard and the central obstacle. Failure to do so triggered an unpleasant sound played through the HMD’s headphones, providing immediate negative feedback.

#### Experimental conditions

Three conditions were used to assess participants’ ability to learn and adapt to the spatial probabilities of the virtual security guard’s appearance: a control condition, a left-biased condition, and a right-biased condition. In the control blocks, the guard had a 50/50 chance of blocking either the left or right pathway in each trial, serving as a baseline measure. To examine adaptive learning, we included two types of biased conditions. In the *left-biased condition*, the guard had an 88% probability of blocking the left pathway, encouraging participants to adapt by predictively adjusting their movement towards the right side. Similarly, in the *right-biased condition*, the guard blocked the right pathway with an 88% probability, prompting participants to adapt by predictively adjusting their movement towards the left side. Participants were not informed about the block type (control, left-biased, right-biased) and the onset of a new block did not provide any information about the upcoming spatial structure. Thus, the probabilistic environment was unpredictable across blocks and had to be newly inferred from experience within each block.

#### Procedure

Participants arrived in the VR lab and the task was explained to them. After verbally agreeing to the task, the participants signed an informed consent form and completed the EHI. Once it was confirmed that they understood the task, they were introduced to the VR equipment, including the headset and controller, and were shown how to operate them. Once the participant was ready, they were moved to the starting position and put on the headset. The experiment began with an eye-tracking calibration procedure. Once the calibration was successfully completed, a short practice session was initiated to familiarize participants with the virtual environment, VR controls, and overall task structure. This session consisted of a shortened version of a control block, comprising 10 trials. After the practice session, participants were asked whether they had any remaining questions before proceeding to the main experiment. Each trial began when the participant pressed a button on the VR controller, which removed a black square displaying the message “Start New Trial” in front of them. Participants then began walking towards one of the two exit doors, attempting to avoid both the central obstacle and the security guard. If their chosen direction was blocked by the guard, they were required to switch to the alternative pathway. After completing a trial, participants returned to the starting position to initiate the next one. The experiment used a within-subjects design with a blocked structure comprising nine blocks with each block being one of the three conditions (three left-biased conditions, three right-biased conditions, and three control conditions), each containing 20 trials. The sequence of blocks was randomized for each participant to control for order effects. The total duration of the experiment was approximately 90 min, including instructions and the practice session. Participants were allowed to take breaks between blocks if needed.

#### Pre-processing and analysis

The following section outlines the preprocessing and analysis procedures for extracting key behavioral and gaze-related metrics. These steps provided the foundation for assessing participants’ movement strategies, learning effects, and gaze behavior across trials and experimental conditions, as reported in the results section. All pre-processing and data analysis were done in Python 3.12.3, with R-based mixed-effects models (lme4, nlme) accessed through Python via pymer4. Full model tables for all mixed-effect analyses are provided in the Supplemental Information.

*Body movement data. *To examine potential predictive movement adjustments, we focused on the participant’s last x-position (left vs. right) immediately before the security guard appeared. Position data were sourced from the waist tracker to capture lateral movement adjustments made in anticipation of the virtual security guard before it actually became visible. To analyze behavior across left-biased and right-biased conditions, we normalized the x-position data by aligning all trials such that movements towards the unblocked pathway were coded as positive values. In other words, we flipped the x-position values for trials in which the likely unblocked pathway was on the left, ensuring that, in both cases, anticipatory movement towards the likely unblocked side was reflected as a shift in the same direction (positive x). The value of the normalized x-position thus indicated how strongly participants adjusted their movement towards the anticipated unblocked path before the security guard appeared, with more positive values reflecting stronger anticipatory shifts in the unblocked direction. Importantly, this analysis was not based on a fixed ideal trajectory, such as a straight line down the center, but instead captured directional biases relative to what would be expected if participants made no consistent anticipatory adjustments. That is, if lateral deviations towards the blocked and unblocked sides occurred at random, they would average out over trials. By normalizing the data in this way, we were able to detect systematic, directional movements towards the likely unblocked side, which we interpreted as evidence for adaptive adjustment based on acquired knowledge throughout the block.

For visualization of movement trajectories, we extracted time-series position data for each trial and focused on participants’ lateral (x-axis) position as they progressed through the corridor (z-axis). To ensure comparability across trials of varying duration and speed, we interpolated each trajectory’s x-positions at a fixed set of predefined z-axis positions. This approach allowed us to compare lateral movement at equivalent forward positions across trials and participants. Individual trajectories were plotted alongside average trajectories for early and late trials within each block to assess learning effects over time.

*Eye data. *In addition to examining lateral body movement adjustments, we analyzed participants’ gaze scanning behavior via the HMD’s integrated eye tracker. Gaze data were sampled at 120 Hz, providing gaze-direction coordinates at each timestep. Similarly to the movement data, we focused on gaze data before the appearance of the security guard. To identify fixations and saccades, we employed an I-DT (Dispersion Threshold) algorithm in three dimensions. Consecutive samples were grouped into a fixation if their spatial dispersion remained within a bounding region of 5 cm and if the group persisted for at least 100 ms, which has been commonly established as the minimum duration for a fixation^23^. Samples exceeding these thresholds were classified as part of a saccade. From these classifications, we derived two trial-level gaze-scanning metrics: the saccade rate and fixation dispersion. The saccade rate was calculated as the number of saccades per second, thereby accounting for differences in trial duration. Fixation dispersion quantifies the spatial variability of fixation centers within a trial and was computed as the root-mean-square distance of individual fixation centers from the overall centroid of fixations. This measure reflects the degree of focus in participants’ gaze: lower dispersion indicates a more concentrated, stable gaze pattern, while higher dispersion suggests a more scattered scanning strategy.

### Results

#### Lateral movement bias in the control condition

To determine whether participants exhibited an inherent lateral movement bias regardless of condition or block, we analyzed their behavior in the control condition. The control blocks, in which the guard appeared with equal probability on the left and right, served as a baseline, allowing us to assess if participants consistently favored one side. We calculated each participant’s average last x-position before the security guard appeared across all control trials and conducted a one-sample t-test against zero. The analysis indicated no significant lateral bias in the control condition, *t*(28) =  −1.47, *p* = 0.154, with a mean of −0.03 m (SD = 0.12, 95% CI [−0.08, 0.01]), suggesting that participants did not show a consistent preference for left or right walking paths.

We next tested whether participants showed trial-by-trial adjustments within control blocks. A mixed-effects linear model predicting normalized last x-position from trial number within block showed no evidence of a systematic trial-to-trial change (β = −0.001, SE = 0.005, *p* = 0.803, 95% CI [−0.010, 0.008]). This indicates that, when no predictive environmental regularities were present, participants’ lateral movement patterns remained stable across trials.

#### Trial-by-trial learning within experimental blocks

**Fig. 2:**
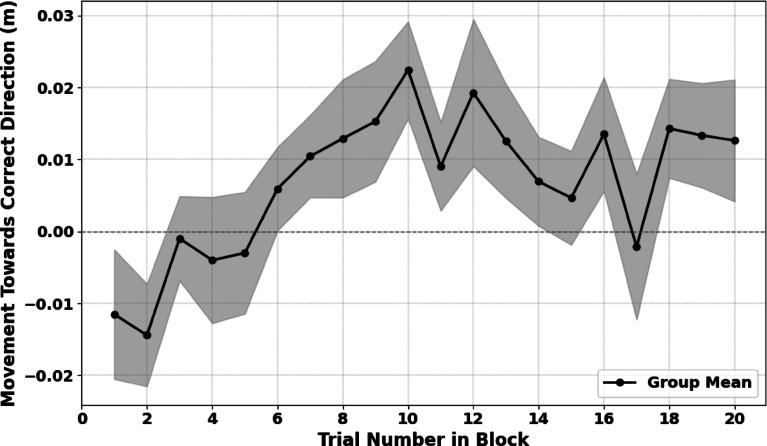
Trial-by-trial movement adjustments in Experiment 1. The plot shows the average normalized x-position before security guard appearance, recorded such that positive values indicate movement towards the likely free direction. The shaded area represents the standard error of the mean across participants.

Visual inspection of participants’ trial-by-trial lateral movement adjustments before security guard appearance (see Fig. 2) suggested an exponential pattern: participants quickly learned to anticipate the guard’s position in early trials, with improvement tapering off in later trials. To statistically test for an exponential learning effect, we fitted a mixed-effects linear model predicting normalized last x-position from an exponential-decay transformation of trial number within block (exp(-trial)), where higher values indicate early trials, and values near zero represent later trials. Rather than fitting a fully nonlinear exponential model, we chose this approach to retain the flexibility of linear mixed modeling—allowing for random slopes and additional predictors—while still capturing the expected shape of learning. Importantly, it also facilitated more straightforward interpretation of model parameters and comparison across models. The model included participant as a random intercept to account for baseline position differences and allowed us to test whether participants increasingly shifted their movement towards the likely free pathway across trials within left- and right-biased blocks.

The fixed-effect slope for the exponential-decay term was negative and significant, *β* = −0.18, *SE* = 0.05, *z* = −3.89, *p* < 0.001, 95% CI [−0.27, −0.09], confirming stronger anticipatory adjustments in later trials compared to earlier ones. However, the standardized effect size was modest (standardized *β* = − 0.06), despite the exponential model explaining substantial variance at the group level (marginal *R*^*2*^ = 0.40). The conditional *R*^*2*^ (0.42) was only slightly higher, indicating that individual baseline differences alone explained minimal additional variance. Given substantial individual differences suggested by single-subject plots, we expanded the model by adding random slopes for the exponential-decay predictor. In this richer model, the exponential effect remained significant (*β* = −0.18, *SE* = 0.08, *z* = −2.09, *p* = 0.036, 95% CI [−0.34, −0.01]), but the conditional *R*^*2*^ increased notably to 0.79, indicating substantial between-subject variability in how strongly participants adapted to the environmental regularities of guard appearance. Examination of random slopes showed that few individuals exhibited steep negative slopes (i.e., stronger, faster adaptation), while most had near-zero or even positive slopes, indicating minimal or no change in behavior over trials. To illustrate the practical magnitude of the observed effect, we compared the model’s predicted lateral adjustment before the security guard appeared in the first versus last trial of a block. According to the model, participants shifted their movement trajectory towards the likely free pathway by approximately 0.065 m (95% CI [0.032 m, 0.097 m]) from the first to the last trial within a block, demonstrating a measurable though modest learning effect on the group level. Given that the lateral distance between the starting point and the exit doorknob was 1.5 m, and the central obstacle blocked 0.85 m of the museum’s middle section, this shift reflects only a small fraction of the lateral movement adjustment required to successfully reach the likely free exit door.

#### Learning across experimental blocks

Next, we tested whether learning effects extended beyond individual blocks, reflecting cumulative behavior changes across the experiment. To do this, we fitted a mixed-effects linear model predicting lateral adjustments based on trial number within block, block position (order of biased blocks, excluding controls), and their interaction, while participant was included as a random effect, allowing individual differences in baseline positions and learning slopes. The main effect of block position was not significant (*p* = 0.261), indicating that participants’ overall lateral position before security guard appearance did not systematically differ across blocks. Likewise, the interaction between block position and trial-based learning was not significant (*p* = 0.323), suggesting the magnitude or rate of within-block learning did not systematically differ between earlier and later blocks. Interestingly, once block position was included, the exponential-decay effect of trial number within block — which previously explained substantial group-level variance (marginal *R*^*2*^ = 0.40) — was no longer significant (*p* = 0.489), and the marginal *R*^*2*^ sharply decreased to 0.15. This dramatic drop in explained group-level variance suggests that the previously observed exponential learning effect primarily reflected trial-by-trial adjustments occurring independently within each block rather than cumulative learning across the experiment as a whole. However, the conditional *R*2 remained high (0.77), again underscoring the substantial individual differences: some participants showed robust adaptation within blocks, whereas others exhibited minimal or inconsistent adjustment. Thus, although learning occurred within individual blocks, primarily driven by a small subset of adapting individuals, we found no reliable evidence for additional improvement across blocks at the group level.

#### Individual differences in movement strategies and cluster analysis

The substantial between-subject variability indicated by our mixed-effects models prompted us to further explore individual participant trajectories. Single-subject trajectory plots confirmed that only a minority consistently made noticeable predictive adjustments in their walking direction. In contrast, most participants maintained a straight walking trajectory, adjusting laterally only after the security guard became visible. This qualitative inspection aligns with our statistical findings, further highlighting that the modest group-level learning effect primarily reflects the behavior of a small subset of adapting individuals rather than widespread anticipatory learning across all participants.

**Fig. 3:**
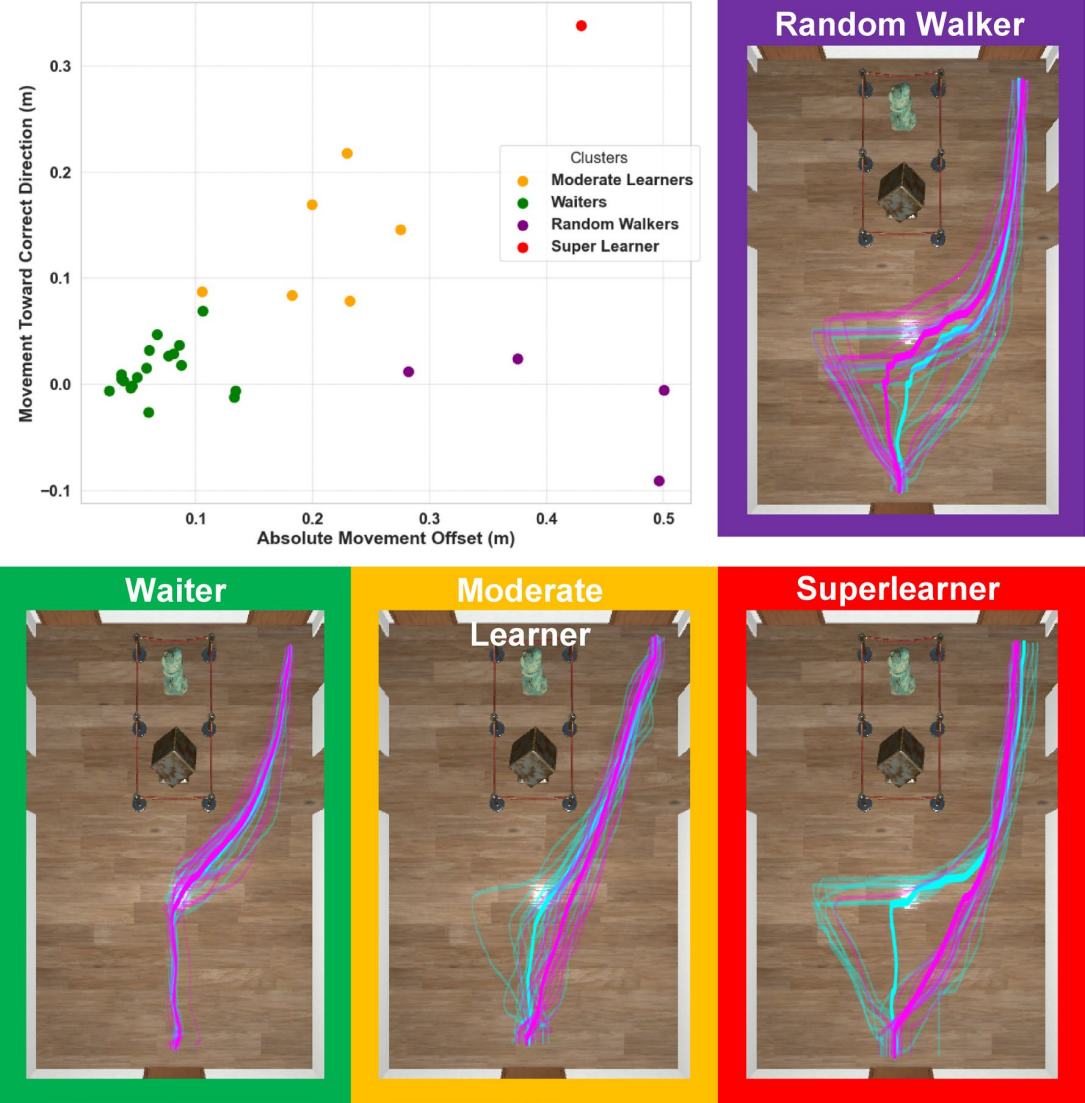
Cluster analysis of movement strategies and representative participant trajectories. Top left: k-means clustering of participants based on their average lateral offset and offset towards the correct direction (i.e., away from the blocked path) before guard appearance in the last five trials of each biased block. The four resulting clusters reflect distinct behavioral strategies: Waiters, Moderate Learners, Random Walkers, and Super Learners. Right and bottom: Example trajectory plots from one representative participant per cluster, illustrating typical movement patterns in the first five and last five trials of *right-biased blocks* (i.e., blocks where the guard appeared on the left side). Thin blue lines represent individual trial trajectories from the first five trials; the bold blue line indicates their average. Thin pink lines represent individual trajectories from the last five trials; the thick pink line shows their average.

Building on these observations of individual differences, we conducted a two-dimensional k-means cluster analysis to categorize participants into four distinct groups based on their movement strategies: Waiters, Moderate Learners, Super Learners, and Random Walkers. These cluster labels reflect observable movement strategies, not underlying cognitive states. For example, participants classified as Waiters may well have learned the regularities but still chose to delay their movement adjustments until the guard became visible. Four clusters were chosen based on interpretability and the behavioral distinctiveness of the resulting subgroups. Both metrics for this clustering were derived from the x-position measured immediately before the security guard appeared. Specifically, we considered (1) the average movement offset, defined as the absolute magnitude of lateral deviation from a straight trajectory, regardless of direction and (2) the average movement towards the correct direction (lateral shifts aligned with the likely unblocked pathway). We selected these two metrics because they capture the central components of predictive movement adjustments in our task. Together, they reflect how strongly participants deviated laterally from a straight path and how much this deviation aligned with the more likely unblocked pathway. Because the second metric more directly captures strategic, probability-based adjustments rather than random lateral variation, we explored different weightings of this measure (1.0, 1.5, and 2.0). The final analysis used a weighting of 2.0, as lower values caused a small number of participants without anticipatory adjustments to merge with Moderate Learners despite their qualitatively different behavior. While subgroup structure was largely stable across weightings, the value of 2.0 offered a slightly clearer distinction between predictive and non-predictive strategies. To focus on learned behavior, we only considered the last five trials of each left- or right-biased block, assuming that participants would have had sufficient exposure by then to anticipate the guard’s likely position. This clustering procedure yielded discrete groups reflecting distinct movement strategies. Using these groups for classification, the majority of participants were classified as Waiters (n = 18), making minimal lateral movements and essentially waiting until the security guard’s position was apparent before adjusting. A smaller subset of Moderate Learners (n = 6) demonstrated some predictive adjustment, although their lateral shifts were rather small, and Random Walkers (n = 4) exhibited inconsistent lateral shifts that did not reliably match the guard’s position. Only one participant emerged as a ‘Super Learner,’ displaying robust, predictive lateral adjustments towards the open pathway after the first few trials. This individual combined high lateral offsets with accurate alignment to avoid the guard, suggesting a strongly anticipatory strategy based on the guard’s likely location. To visually illustrate the diversity of movement strategies, Fig. 3 shows the results of our cluster analysis and representative movement trajectories from one participant in each cluster.

**Fig. 4:**
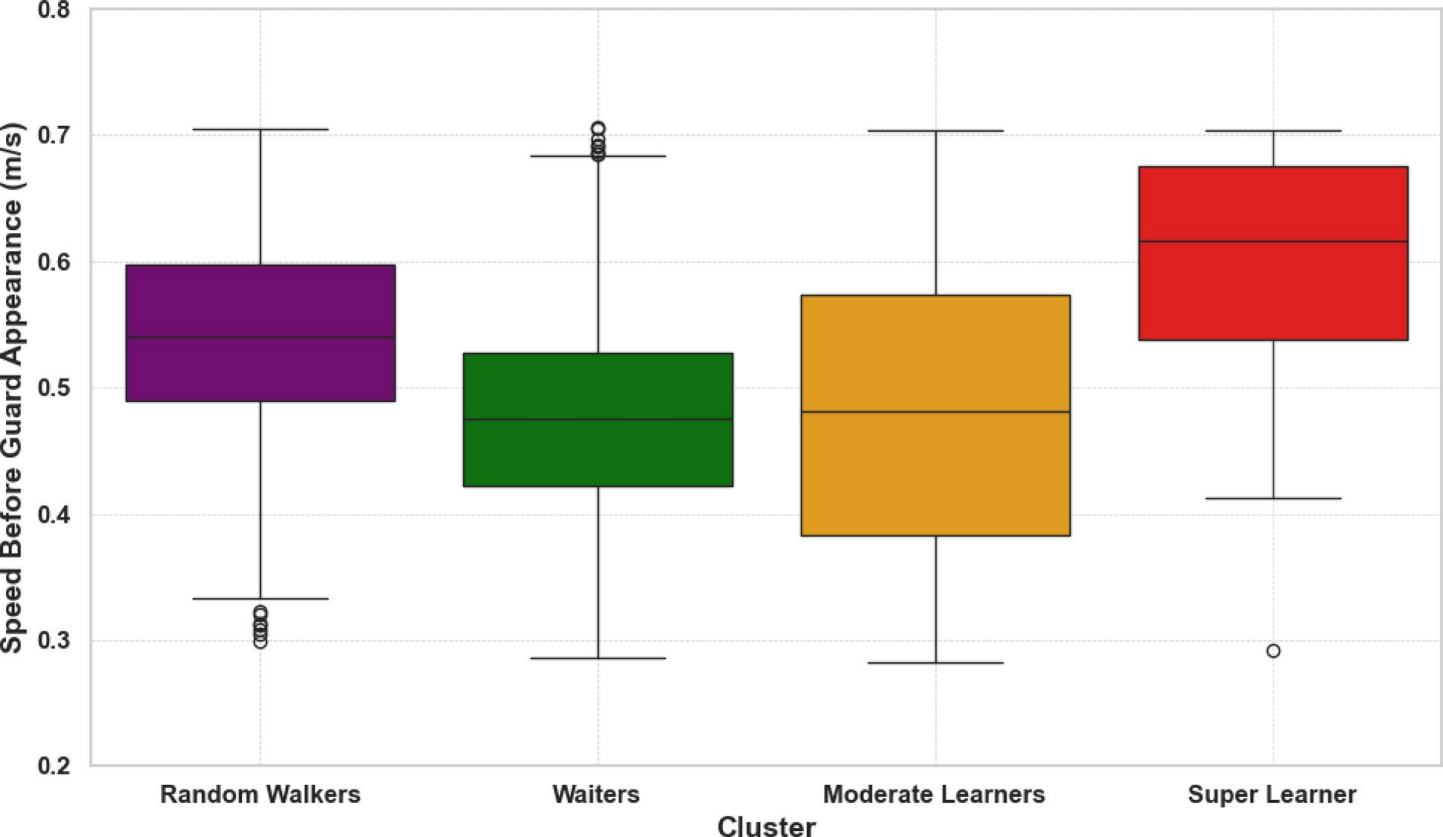
Walking speed before security guard appearance by cluster. The boxplots show the distribution of walking speeds before the appearance of the security guard, grouped by the four participant clusters: Random Walkers, Waiters, Moderate Learners, and Super Learner. The Waiters group displayed significantly slower speeds compared to members of the other three clusters, reflecting a strategy of waiting for visual confirmation of the guard’s position.

Notably, an analysis of walking speed before guard appearance confirmed that Waiters indeed “waited” more than members of the other three clusters: a mixed-effects model revealed significantly lower speeds for Waiters (*β* = −0.07, *SE* = 0.02, *z* = −3.02, *p* = 0.003, 95% CI [−0.11, −0.02], standardized *β* = −0.25), consistent with a strategy of slowing down before being able to visually confirm the guard’s position. Figure 4 illustrates the distribution of walking speeds across clusters. Overall, these findings indicate that most individuals relied heavily on visual confirmation rather than preemptively performing adjustments according to learned probabilities.

The prevalence of delayed lateral adjustments can also be interpreted in light of the environment’s spatial layout. Using a fixed commitment threshold of ±0.25 m to classify trials, path length differed significantly across categories (Kruskal–Wallis *H* = 16.45, *p* < 0.001). Early movement towards the incorrect pathway resulted in substantially longer routes (0.68 m more than trials without early commitment), whereas correct early commitments provided comparatively small savings relative to walking close to the midline (0.09 m).

#### Gaze behavior

In addition to lateral body movement adjustments, we also examined participants’ gaze scanning behavior prior to the security guard’s appearance, focusing specifically on saccade rate and fixation dispersion, using mixed-effects models. Each model included trial number within block (to measure within-block adaptation), condition (biased vs. control blocks), block number (reflecting changes across the whole experiment), and relevant interaction terms. These analyses enabled us to distinguish changes occurring within individual blocks from broader, experiment-wide adaptations.

For saccade rate, participants scanned significantly more frequently in control blocks compared to biased blocks (*β* = 0.17, *SE* = 0.05, *z* = 3.39, *p* = 0.001, 95% CI [0.07, 0.27], standardized *β* = 0.09). Additionally, saccade rate decreased significantly as the experiment progressed (*β* = −0.05, *SE* = 0.01, *z* = −5.69, *p* < 0.001, 95% CI [−0.07, −0.03], standardized *β* = −0.16), indicating reduced visual exploration with increasing familiarity. Neither trial number within block (*p* = 0.063) nor its interactions with condition (*p* = 0.307) and block number (*p* = 0.103) were significant.

In the analysis for fixation dispersion, we observed significant decreases both within individual blocks (*β* = −0.011, *SE* = 0.003,* z* = −4.36,* p* < 0.001, 95% CI [−0.016, −0.006], standardized *β* = −0.14) and across the experiment (*β* = −0.04, *SE* = 0.01, *z* = −7.62, *p* < 0.001, 95% CI [−0.05, −0.03], standardized *β* = −0.23). This suggests that participants’ gaze became progressively more focused as they gained familiarity with the environment. A small but significant interaction between block number and trial number (*β* = 0.001, *z* = 3.29, *p* = 0.001, 95% CI [0.001, 0.002], standardized *β* = 0.13) indicated that the reduction in fixation dispersion within each block became slightly weaker in later blocks. However, neither condition (*p* = 0.416) nor its interaction with trial number (*p* = 0.999) significantly influenced fixation dispersion.

Despite statistical significance, the practical impact of these effects was limited, as fixed effects explained only a very small portion of the total variance (marginal *R*^2^ between 0.02 and 0.03). In contrast, conditional *R*^2^ values were notably higher (0.33 for saccade rate; 0.19 for fixation dispersion), underscoring substantial individual variability. Taken together, these findings suggest that although participants slightly adjusted their gaze scanning as they became more familiar with the environment, these adjustments were rather limited. Instead, individual differences in gaze patterns and strategies appeared to be more influential.

### Discussion

The present study aimed to investigate whether participants are able to learn and anticipate the likely location of an upcoming obstacle, a virtual security guard, based on repeated exposure to probabilistic environmental regularities. Specifically, we hypothesized that participants would adjust their movement trajectories predictively, shifting their walking direction towards the likely open pathway before the obstacle became visible. In addition, we explored whether changes in gaze behavior—such as reduced visual scanning or more focused fixations—would reflect increasing certainty about the guard’s location over time.

Our findings revealed a high individual variability in participants’ movement strategies. Only one participant consistently demonstrated large anticipatory adjustments that aligned with the spatial probability of the security guard’s appearance, indicative of a fully anticipatory strategy. By contrast, the majority of participants did not exhibit such early adjustments; most adopted a ‘waiting’ strategy, maintaining a straight trajectory until the security guard became visible, thereby delaying their lateral movement decision. In addition, while a subset of participants (categorized as Moderate Learners) showed some degree of predictive adjustment, another group (Random Walkers) exhibited large lateral movements that did not correspond to the spatial probabilities of the guard’s appearance.

The prevalence of the waiting strategy does not necessarily reflect a failure to learn; rather, it might represent an alternative approach under conditions of uncertainty. Although this strategy necessitates larger corrective adjustments after the guard appears, it reduces the need for internal prediction and planning, thereby minimizing cognitive effort. This is consistent with the soft constraints hypothesis^24^, which proposes that people flexibly trade off cognitive and motor or perceptual effort depending on which strategy minimizes time or resource costs in a given context. More broadly, it aligns with evidence that people systematically avoid cognitive demand when less effortful alternatives are available^25^. In addition, our environment’s spatial layout favored delayed commitment. Pronounced predictive movement adjustments towards the incorrect pathway imposed substantial additional path length, whereas correct early commitments yielded rather small benefits. Therefore, midline “wait-and-see” behavior minimized the risk of considerably longer walking distance. This pattern was also reflected in movement smoothness: by avoiding large corrections arising from early wrong-side commitments, Waiters and Moderate Learners showed small mean deviations from minimum-jerk trajectories (see supplement Sect. 1.6, Figs. S1–S2).

To explore whether participants became more certain about the guard’s location over time regardless of their strategy use, we looked at changes in their gaze behavior. Our analyses revealed significant but modest reductions in visual scanning (saccade rate) and progressively narrower fixation dispersion both within and across experimental blocks. These results suggest increased familiarity and reduced uncertainty regarding the likely location of the guard’s future appearance over time. However, the practical impact of these gaze adjustments was limited, given the minimal variance explained at the group level and substantial individual differences.

To test whether participants were actually capable of learning and adapting their behavior to the environment’s regularities, we designed a follow-up experiment in which we eliminated the possibility of a waiting strategy. This change also introduced greater behavioral costs for incorrect choices, as participants who selected the blocked pathway would have to walk back and start over, rather than simply making a lateral movement adjustment. By enforcing early pathway selection and increasing the cost of incorrect choices, we aimed to determine whether participants could learn and adapt to the spatial probabilities of the appearance of the security guard without relying on the waiting strategy.

## Experiment 2

Following the findings of Experiment 1, we designed a follow-up experiment to determine whether participants would learn the environment’s spatial regularities when the waiting strategy was no longer viable. This allowed us to explore whether the absence of predictive adjustments in the Waiter subgroup reflected a failure to learn or simply a different strategy.

### Methods

**Fig. 5:**
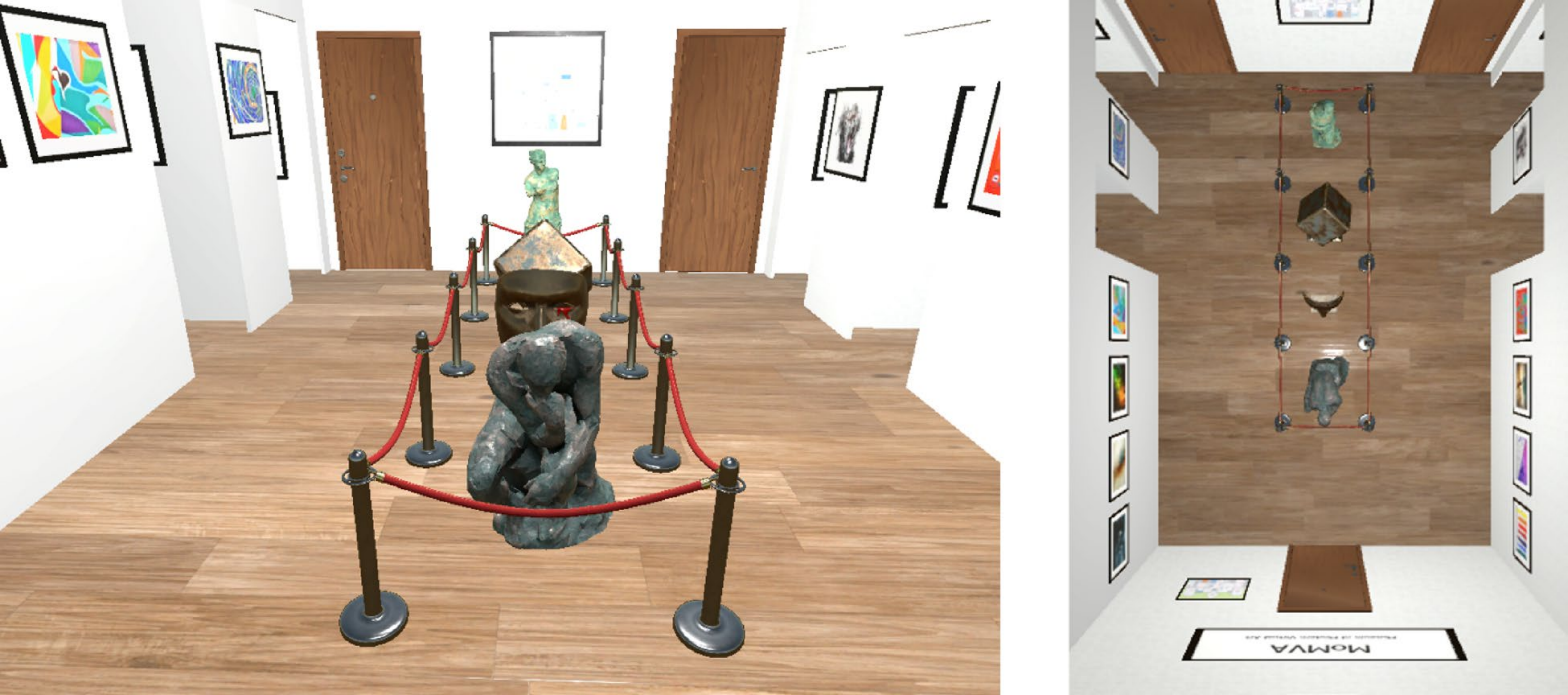
Virtual environment layout in Experiment 2. This figure illustrates the design change regarding the central obstacle, which forced participants to make a definite pathway decision early on. The pathways were separated by 1.2 m from the starting position, and the security guard appeared within a range of 1.2 to 1.6 m. If participants initially chose the blocked path, they had to walk back and then proceed along the alternative pathway.

A new sample of 25 participants (mean age 25.88 years, *SD* = 3.85, range = 20–35 years, 23 right-handed) completed Experiment 2. The experimental setup and task procedure were as in Experiment 1, with two main adjustments. First, the pathways were separated already 1.2 m from the start position, and the appearance of the security guard was triggered slightly later (within a range of 1.2 to 1.6 m from the start position)—forcing participants to make an earlier decision on which path to take. Second, due to the increased time required for trials where participants chose the blocked pathway and needed to walk back, one of the three control blocks was removed to keep the experiment duration manageable. Figure 5 shows the new layout of the virtual museum from Experiment 2, illustrating the design change regarding the central obstacle. Unlike in Experiment 1, where learning could be inferred from subtle, graded lateral movement adjustments, the design of Experiment 2 required participants to commit to a pathway before the museum guard appeared. This eliminated the option to wait or make only small adjustments. As a result, participants’ lateral offsets were consistently larger — either towards the correct or incorrect side — making the continuous movement variable less informative about the strength or gradual development of learning. To better capture whether participants had learned the spatial regularities of the environment, we modeled their binary choices (correct vs. incorrect pathway) across trials using logistic mixed-effects models. This approach would not have been suitable in Experiment 1, where lateral movements were often minimal and gradual. In that context, it would have been impossible to classify individual trials as “correct” or “incorrect” without arbitrarily setting a cutoff, making binary choice labels unreliable.

### Results

#### Trial-by-trial learning within experimental blocks

To examine whether participants learned to anticipate the likely location of the security guard under conditions that required an early commitment to a pathway, we analyzed participants’ binary choices (correct vs. incorrect pathway) using logistic mixed-effects models with trial number in block as a predictor. This model structure was selected to match the environmental demands of Experiment 2, where participants had to commit to a pathway early in each trial. We did not include random slopes for trial number in block, as a model with random slopes resulted in a singular fit, suggesting minimal interindividual variability in learning rates. The final model, therefore, captured robust group-level learning trends while accounting for baseline differences between participants via random intercepts.

**Fig. 6:**
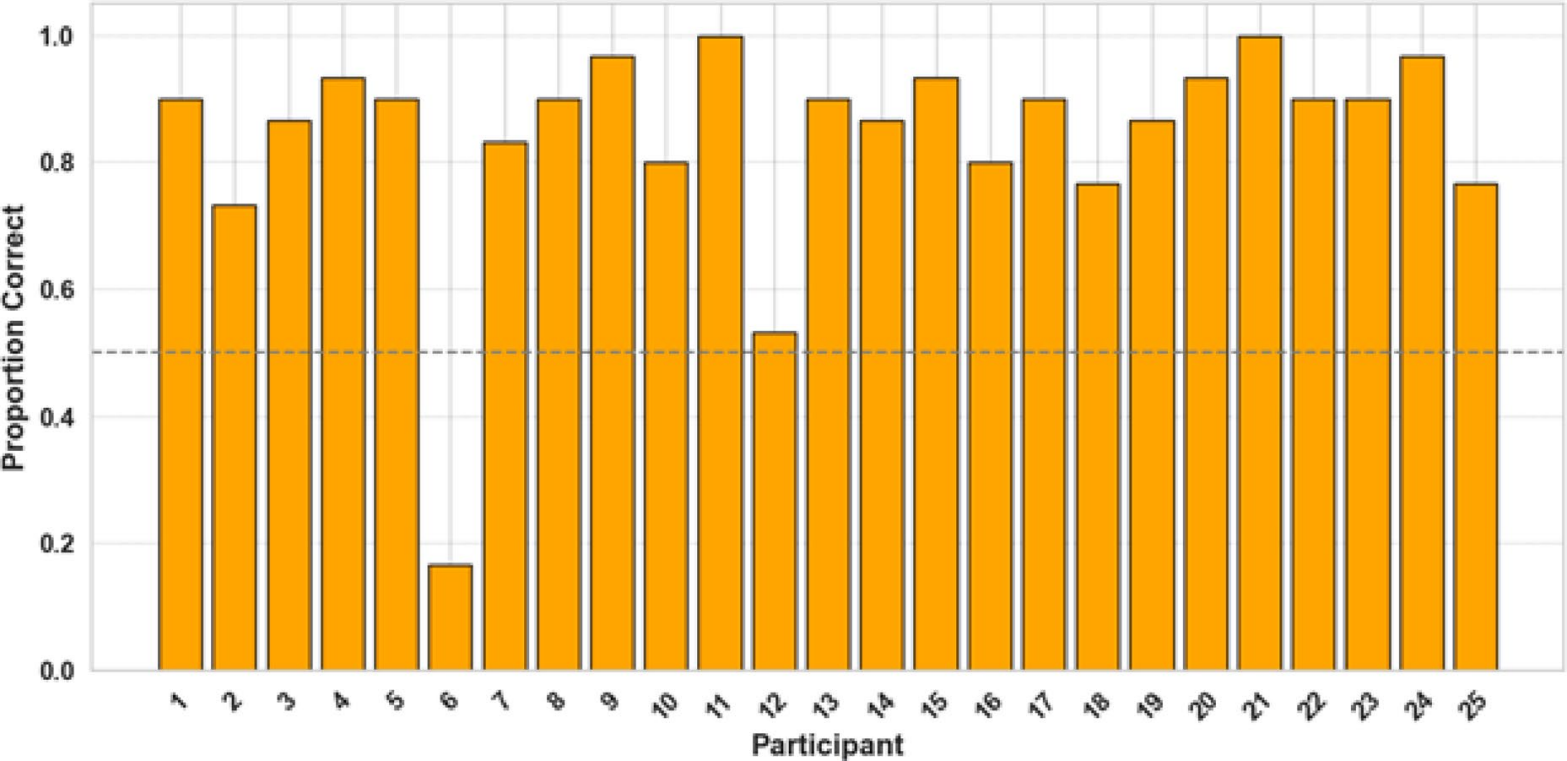
Proportion of correct pathway choices in the final five trials of each biased block for every participant in Experiment 2. The dashed line indicates chance-level performance (50%). Most participants performed well above chance, suggesting reliable learning of the guard’s location.

This was further supported by an analysis of individual subjects’ choice behavior in the final five trials of each biased block. The results revealed that 23 out of 25 participants consistently selected the correct pathway above chance, indicating successful learning of the environmental regularities. One participant performed close to chance, while another showed a surprising deviation with performance well below chance (see Fig. 6).

**Fig. 7:**
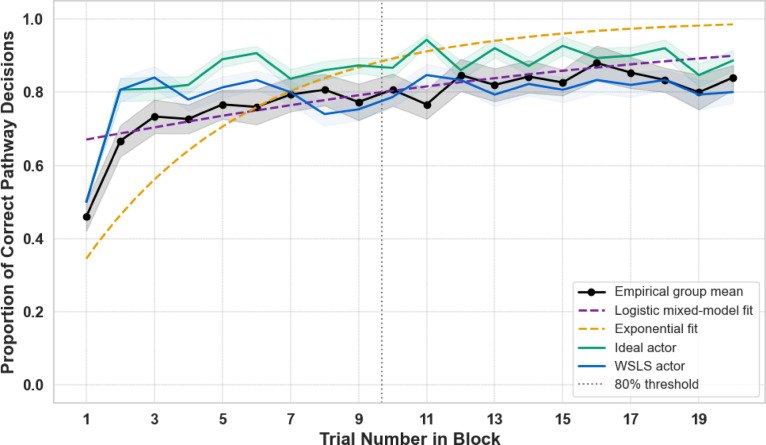
Empirical learning curve across trials in biased blocks, shown together with logistic and exponential model fits and two computational benchmark actors. Black points indicate the mean proportion of correct pathway decisions across participants (±SEM). The purple dashed line shows the logistic mixed-model fit and the orange dotted line the exponential fit. The green line represents a Bayesian ideal actor, and the blue line a win–stay/lose–shift (WSLS) actor exposed to the same trial sequences. The vertical dotted line indicates the trial at which the logistic model predicts that the group reaches 80% correctness. Participants rapidly approached near-optimal performance and more closely resembled the ideal actor than WSLS, particularly in the progression of learning rather than in the final performance level, consistent with block-wise probabilistic learning rather than simple choice repetition.

We also explored an exponential decay model to capture the shape of the learning curve. While it visually provided a better account of the steep accuracy increase during the first few trials, it consistently overestimated performance in later trials (see Fig. 7) and had a higher AIC than the logistic model (2841 vs. 2811). Given the logistic model’s better overall fit, we report its results below.

The logistic mixed-effects model revealed a significant effect of trial number within block on participants’ binary choices, indicating robust trial-by-trial learning. As participants completed more trials within a block, they became increasingly likely to choose the correct pathway (*β* = 0.45, *SE* = 0.05, *z* = 9.12, *p* < 0.001, 95% CI [0.35, 0.55], standardized *β* = 0.45).

This effect indicates that participants gradually learned where the security guard was most likely to appear and adjusted their movement direction accordingly. In contrast to Experiment 1, where predictive behavior was more variable and inferred from subtle lateral movement shifts (*β* = −0.06), participants in Experiment 2 showed clear and consistent learning through their pathway choices.

To benchmark performance against an optimal strategy, we compared participants’ choices to an ideal Bayesian learner-actor that was exposed to the exact same trial-sequences of open pathways, inferred the block-wise probabilistic regularity using Beta-Bernoulli updates from a uniform prior, and selected the pathway with the higher posterior mean probability. For brevity, we refer to this model as the ideal actor. As shown in Fig. 7, the empirical learning curve closely matched the ideal actor benchmark (Pearson *r* = 0.88, RMSE = 0.09), demonstrating that participants extracted and used the block-wise regularities with high efficiency. Performance in late trials approached near-optimal levels (0.83 for participants vs. 0.89 for the ideal actor), reflecting strong, rapid learning under the early-commitment demands of Experiment 2. To rule out the possibility that performance was driven by simple short-term repetition rather than probabilistic learning, we additionally compared the empirical curve to a win–stay/lose–shift (WSLS) actor. Although WSLS reached a similar accuracy plateau due to the strong block-wise bias, it did not reproduce the empirical learning dynamics, with a substantially weaker correspondence to participants’ performance (Spearman *ρ* = 0.23, *p* = 0.34) than for the ideal actor (Spearman *ρ* = 0.48, *p* = 0.03). This dissociation shows that participants’ performance is unlikely to be explained by trial-to-trial repetition alone, but instead reflects the extraction and use of block-wise probabilistic structure.

To verify that learning was specific to the biased blocks, we also examined the control condition, in which the guard appeared equally often on the left and right. In the control blocks of Experiment 2, participants performed at chance level (mean correctness = 0.51, bootstrap 95% CI [0.48, 0.54]), and trial number within block did not predict performance (*β* = −0.09, *SE* = 0.06, *p* = 0.172, 95% CI [−0.21, 0.04]; see supplement Section 2.2, Fig. S3).

#### Pathway selection across experimental blocks

After establishing robust trial-by-trial learning within blocks, we next tested whether participants’ ability to select the correct pathway improved cumulatively across the experiment. To investigate this, we fitted a logistic mixed-effects model including trial number within block, block position, and their interaction as fixed effects. The model revealed a significant main effect of block position (*β* = 0.22, *SE* = 0.05, *z* = 4.39, *p* < 0.001, 95% CI [0.12, 0.31], standardized *β* = 0.22), indicating that participants were generally more likely to make correct decisions in later blocks. However, the interaction between trial number and block position was not significant (*β* = −0.06, *SE* = 0.05, *z* = −1.11, *p* = 0.269, 95% CI [−0.15, 0.04]).

#### The time course of learning within blocks

To further quantify how quickly participants learned the environmental regularities, we used the logistic mixed-effects model described above to estimate the trial number at which predicted accuracy exceeded 80% within a block. Based on the model’s fixed effects, participants reached this threshold by trial 9, on average, indicating that stable learning typically emerged during the first half of each 20-trial block. This reinforces the earlier finding of robust and relatively rapid trial-by-trial learning under the modified conditions of Experiment 2, as shown in Fig. 7, where the 80% accuracy threshold is indicated by the dotted vertical line.

#### Increased walking speeds

Furthermore, removing the waiting strategy also influenced participants’ walking speeds. A Welch’s two-sample *t*-test comparing participant-level mean speeds prior to the guard’s appearance revealed significantly faster speeds in Experiment 2 (*M* = 0.62 m/s, *SD* = 0.08, *n* = 25) relative to Experiment 1 (*M* = 0.50 m/s, *SD* = 0.07, *n* = 29), *t*(48.2) =  −5.91, *p* < 0.001, Cohen’s *d* = 1.60).

#### Gaze behavior

To investigate gaze behavior in Experiment 2, we again used mixed-effects models identical to those employed in Experiment 1, focusing on saccade rate and fixation dispersion as our dependent measures. Each model included trial number within block, condition (biased vs. control), block number, and relevant interaction terms as fixed effects, with participant as a random intercept. Participants’ saccade rates significantly declined across blocks (*β* = −0.04, *SE* = 0.01, *z* = −5.70, *p* < 0.001, 95% CI [−0.06, −0.03], standardized *β* = −0.23), suggesting reduced visual exploration as participants gained familiarity with the environment. However, neither condition (*β* = 0.03, *p* = 0.515, 95% CI [−0.05, 0.10], standardized *β* = 0.03) nor trial number within block (*β* = −0.01, *p* = 0.134, 95% CI [−0.01, 0.00], standardized *β* = −0.06), nor any of their interactions (all *p*s ≥ 0.099), significantly influenced saccade rate. Although the main effect of block number was significant, fixed predictors explained only a small portion of the total variance (marginal *R*^2^ = 0.03). By contrast, the conditional *R*^2^ was notably higher (0.17), indicating substantial individual variability in baseline visual scanning rates. We observed significant decreases in fixation dispersion within individual blocks (*β* = −0.02, *SE* = 0.01, *z* = −3.74, *p* < 0.001, 95% CI [−0.03, −0.01], standardized *β* = −0.16), as well as across the experiment (*β* = −0.08, *SE* = 0.01, *z* = − 7.15, *p* < 0.001, 95% CI [−0.11, −0.06], standardized *β* = −0.28), consistent with increased visual focus as participants became more experienced. Additionally, a small but significant interaction between block number and trial number emerged (*β* = 0.002, *SE* = 0.001, *z* = 2.43, *p* = 0.015, 95% CI [0.000, 0.004], standardized *β* = 0.13), suggesting a slight decrease in the within-block reduction of fixation dispersion in later blocks. Neither condition (*β* = 0.02, *p* = 0.721, 95% CI [−0.10, 0.14], standardized* β* = 0.01) nor its interaction with trial number (*β* = 0.002, *p* = 0.688, 95% CI [−0.008, 0.012], standardized *β* = 0.02) significantly influenced fixation dispersion. As with saccade rate, fixed effects explained only a modest amount of total variance (marginal *R*^2^ = 0.05), while individual differences accounted for substantially more (conditional *R*^2^ = 0.20). Together, these results align closely with Experiment 1, indicating modest group-level adaptations in gaze behavior coupled with substantial individual differences. Participants generally narrowed their gaze and reduced visual scanning across trials and blocks, reflecting increased familiarity rather than strong, condition-specific adaptations.

### Discussion

The goal of Experiment 2 was to directly follow up on the findings of Experiment 1 and test whether participants would learn the environment’s spatial regularities when the waiting strategy was no longer an option. By modifying the setup to require earlier movement decisions and increase the behavioral cost of incorrect choices, we created conditions that more strongly incentivized predictive behavior. Under these constraints, the vast majority of participants (23 out of 25) consistently learned to choose the correct pathway, reflecting a clear understanding of the spatial probabilities present in the environment. This finding aligns with our hypothesis that, once delaying the decision was no longer viable, participants would learn and use the environment’s spatial regularities, demonstrating that they successfully extracted these regularities on a block-by-block basis. Consistent with this interpretation, participants’ learning performance in Experiment 2 closely approximated an ideal Bayesian actor exposed to the same trial sequences, both in overall accuracy and in the shape of the learning curve. Once early commitment was required, participants used the environmental regularities with near-optimal efficiency. This rapid convergence within each block also helps to explain why we did not observe a trial × block interaction, as learning reached a high level so quickly that there was little opportunity for additional acceleration in later blocks. Importantly, this pattern is unlikely to be explained by short-term repetition alone. A win–stay/lose–shift actor reached a comparable accuracy plateau due to the strong block-wise bias, but failed to reproduce the learning dynamics observed in participants. The correspondence between participants’ choices and the WSLS actor was markedly weaker than for the ideal actor, indicating that performance reflected block-wise probabilistic learning rather than local reinforcement from the immediately preceding trial.

Eye-tracking analyses revealed subtle but consistent adaptations in visual scanning behavior across the experiment. As Experiment 2 progressed, participants exhibited reduced saccade rates and increasingly focused fixation patterns (lower fixation dispersion), both within individual blocks and cumulatively over time. However, these adaptations were modest in magnitude and were not reliably influenced by experimental condition (biased vs. control), suggesting that the observed changes in visual scanning were general rather than specifically tied to learning the guard’s probabilistic location. Instead, these general gaze adjustments likely reflect increasing overall familiarity and efficiency in navigating the environment, rather than direct evidence that participants reduced visual exploration because they had learned specific spatial probabilities. Thus, while gaze behavior clearly changed over time, our analyses did not support the conclusion that visual scanning patterns directly reflected participants’ learned expectations about spatial probabilities.

While most participants in Experiment 2 quickly adapted to the environmental regularities, a small minority (2 out of 25) continued to choose pathways randomly, even in later trials of biased blocks. This exception suggests that learning was not entirely universal, though it was highly consistent overall. One possible explanation is variation in executive function. Individuals with stronger executive abilities have been shown to adapt more rapidly in sensorimotor tasks that involve explicit strategic adjustments^26^. Thus, while the results reflect a robust group-level learning effect in movement behavior, they also point to small but meaningful individual differences. In contrast, gaze behavior varied substantially between individuals, suggesting that participants used different visual strategies that were less directly related to probabilistic learning.

## General discussion

Our findings demonstrate that under appropriate environmental constraints, participants can flexibly adapt to probabilistic regularities to guide movement decisions. How this knowledge is expressed in behavior, however, depends critically on the structure of the environment. While Experiment 2 revealed clear evidence of predictive motor planning, Experiment 1 showed substantial individual variability, with many participants delaying their decision until the obstacle appeared. This contrast highlights how environmental constraints shape whether regularities are learned and applied through anticipatory movement planning or remain unused in favor of reactive responses to sensory input once uncertainty is resolved.

In Experiment 1, the dominant use of a waiting strategy suggests that many participants preferred to delay commitment even when probabilistic knowledge could have supported predictive motor planning. A contributing factor may have been the asymmetry in path-length outcomes for early lateral commitments. Pronounced early adjustments towards the incorrect side resulted in considerably longer routes, whereas correct early commitments offered comparatively modest distance savings. This asymmetry made delayed commitment a low-risk option and may have reinforced the strategy of waiting until uncertainty was resolved.

Beyond these structural factors, some participants may have learned the regularities but chosen not to act on them due to perceived effort or strategy preference. Related research in navigation contexts shows that individuals trade off cognitive effort against flexibility, with some favoring low-effort, habitual strategies even when more adaptive alternatives are available^27^. This behavior aligns with findings by Onagawa and Kudo^28^, who observed that participants often adopted seemingly suboptimal strategies that prioritized ease or flexibility under time pressure, even when more rewarding alternatives were available.

A complementary explanation is that participants offloaded cognitive demands onto the environment. Ballard et al^29^ showed that people often prefer to visually acquire task-relevant information instead of relying on their memory capacities. In our task, waiting until the obstacle appeared may have served a similar function, allowing participants to bypass the effort of internalizing, retrieving, and applying probabilistic structure. Petitet et al.^30^ further support this idea by showing that selecting and acquiring the most informative samples during information gathering requires considerable cognitive effort.

These behavioral strategies underscore that merely having the opportunity and cognitive capacity to learn is not sufficient for predictive planning; whether such knowledge is acquired and applied depends critically on environmental demands. In Experiment 2, where early commitment was enforced and incorrect choices required backtracking, participants consistently learned and applied spatial regularities with a learning curve closely aligning with an ideal Bayesian actor. In contrast to Experiment 1, they used predictive strategies, demonstrating that such priors can be acquired and applied through experience alone in an immersive, naturalistic environment. This supports the idea that humans can estimate probabilities implicitly through action and perception, consistent with prior evidence for ‘wordless’ statistical learning^31^. The absence of a viable waiting strategy, combined with more salient negative consequences for incorrect choices, likely promoted learning and using the environmental probabilities. Accordingly, Cluff and Scott^32^ found that trajectory corrections are only executed when explicitly required by the task. These findings suggest that participants flexibly regulate their behavior based on the expected utility of anticipatory adjustments.

Individual differences in predictive planning can also be interpreted through the lens of risk sensitivity and strategic flexibility. Wong et al.^33^ argued that intermediate movements are not merely indecisive compromises but deliberate strategies to delay commitment while preserving optionality under uncertainty. Notably, they highlight that while some individuals consistently produce intermediate responses, others rely on direct responses, and some display a mixture of both. In our task, this might explain not only the waiters in Experiment 1, but also the more nuanced behavior of moderate learners who showed minor anticipatory adjustments. Supporting this, Alhussein and Smith^34^ found that intermediate movements under uncertainty represent optimized motor strategies, not noise, and could thus reflect an early and deliberate commitment to a compromise strategy. These findings suggest that what looks like indecision may in fact be an adaptive motor computation.

Taken together, our results demonstrate that predictive motor planning is not determined solely by the availability of probabilistic information. Instead, it emerges from the interplay between learning capacities, the demands imposed by the task, the constraints of the environment, and individual differences in effort sensitivity and risk tolerance. Understanding this interplay is critical for understanding embodied decision-making and motor planning in complex, naturalistic contexts.

Finally, several limitations should be acknowledged. In Experiment 1, the incentives for early commitment were relatively weak, making it difficult to fully distinguish learning from strategy choice. Moreover, our behavioral analyses cannot determine exactly how participants internally represented spatial probabilities. Future work could more directly examine how such probabilistic structures are encoded and used during movement planning in even richer or more complex environments.

## Supplementary Information

Below is the link to the electronic supplementary material.

Supplementary Material 1

## Data Availability

All data (trial information, eye-tracking data, and movement data) and analysis scripts are available on OSF ( [https://osf.io/5r284/?view\_only=51fa8fdc638e4dc38fcc607b56bc6089](https:/osf.io/5r284/?view_only=51fa8fdc638e4dc38fcc607b56bc6089) ).
