# Supplementary material for: Predictive use of environmental regularities requires action relevance
Source: Sci Rep. 2026 Jan 13;16:1596. doi: 10.1038/s41598-026-35500-x (PMC12800049; doi:10.1038/s41598-026-35500-x)
Supplement: Supplementary file 1 — Supplementary Material 1 [file 41598_2026_35500_MOESM1_ESM.pdf]

Supplementary Information: Predictive Use of Environmental Regularities Requires Action Relevance

Benedikt Kretzmeyer[1], Constantin A. Rothkopf[2], Katja Fiehler[1*]

* Corresponding author
Email: Katja.Fiehler@psychol.uni-giessen.de

[1] Experimental Psychology,
Justus Liebig University Giessen
Otto-Behaghel-Str. 10F
35394, Giessen, Germany

[2] Psychology of Information Processing
Technical University of Darmstadt
Alexanderstr. 10
64283 Darmstadt, Germany

1. Experiment 1

1.1. No Trial-by-Trial Movement Adjustments in Control Condition

Table S1. Mixed-Effects Model: Control Condition Movement

Fixed effects

Predictor	β	SE	z/t	p	95% CI low	95% CI high	Std. β
Intercept	0.002	—	—	—	—	—	—
z_trial	-0.001	0.005	-0.25	.803	-0.010	0.008	-0.005

Random effects

Effect	Variance	SD
Participant (Intercept)	0.000	—

Model fit

Marginal R^2 = 0.000
Conditional R^2 = 0.000

1.2. Trial-by-Trial Learning Models

Table S2a – Mixed-Effects Model (Random Intercept)

Fixed effect	β	SE	z/t	p	95% CI low	95% CI high	Std. β
Intercept	0.032	0.011	2.809	.005	0.010	0.054	—
exp(–trial)	–0.176	0.045	–3.887	<.001	–0.265	–0.087	–0.064

Random effects

Effect	Variance	SD
Participant (Intercept)	0.003	0.057

Model fit

Marginal R^2 = 0.395
Conditional R^2 = 0.419

Table S2b – Mixed-Effects Model (Random Intercept + Random Slope)

Fixed effect	β	SE	z/t	p	95% CI low	95% CI high	Std. β
Intercept	0.032	0.013	2.421	.015	0.006	0.058	—
exp(–trial)	–0.176	0.084	–2.092	.036	–0.341	–0.011	–0.064

Random effects

Effect	Variance	SD	Corr. w/ Intercept
Participant (Intercept)	0.005	0.067	—
Participant (Slope exp(–trial))	0.147	0.384	–0.950

Model fit

Marginal R^2 = 0.401
Conditional R^2 = 0.794

1.3. Learning Across Blocks

Table S3. Mixed-Effects Model: Learning Across Experimental Blocks

Fixed effects

Predictor	β	SE	z/t	p	95% CI low	95% CI high	Std. β
Intercept	0.023	0.015	1.47	.141	-0.008	0.053	—
exp(–trial)	-0.086	0.124	-0.69	.489	-0.330	0.158	-0.031
Block position	0.003	0.002	1.12	.261	-0.002	0.007	0.020
exp(–trial) × Block position	-0.026	0.026	-0.99	.323	-0.078	0.026	-0.037

Random effects

Effect	Variance	SD	Corr. w/ intercept
Participant (Intercept)	0.005	0.067	—
Participant (Slope exp(–trial))	0.148	0.385	-0.949

Model fit

Marginal R^2 = 0.152
Conditional R^2 = 0.771

1.4. Walking Speed Differences Between Clusters

Table S4. Mixed-Effects Model: Walking Speed Before Guard Appearance (Waiters vs. Others)

Fixed effects

Predictor	β	SE	z/t	p	95% CI low	95% CI high	Std. β
Intercept	0.538	0.017	30.76	<.001	0.504	0.572	—
C(Waiters)	-0.067	0.022	-3.02	.003	-0.111	-0.024	-0.253
Trial number	0.001	0.000	1.40	.162	-0.000	0.001	0.023

Random effects

Effect	Variance	SD	Corr. w/ intercept
Participant (Intercept)	0.003	0.055	—
Participant (Slope Trial number)	0.000	0.000	—

Model fit

Marginal R^2 = 0.951
Conditional R^2 = 0.961

1.5. Path Lengths by Early Lateral Movement Category

Table S5. Path Lengths by Early Lateral Movement Category

Trial Type	Mean path (m)	SD (m)	n (trials)	n (participants)
Correct pathway	4.90	1.34	620	17
Midline waiting	4.99	0.89	3984	29
Wrong pathway	5.67	0.55	466	14

Kruskal–Wallis test (subject-wise mean path length across trial types): H = 16.45, p < .001.

1.6. Lateral Deviations from Smooth Minimum-Jerk Trajectories

Because forward movement was required to complete the task and did not reflect a free motor decision, minimum-jerk comparisons were restricted to the lateral component of movement (i.e., the dimension in which participants could freely choose how and when to deviate). Minimum-jerk theory[1] applies to the controllable degrees of freedom; in this task, only lateral adjustments reflected different movement strategies. Evaluating minimum-jerk trajectories in 2D would therefore be dominated by non-strategic forward motion and tracking noise and would not meaningfully describe motor planning.

For each trial, we constructed a one-dimensional minimum-jerk trajectory between the observed initial and final lateral positions using the standard minimum-jerk formulation:

$$x\_MJ(t) = x_0 + (x\_T - x_0)(10s^3 - 15s^4 + 6s^5)$$

Observed lateral trajectories were resampled to 200 equally spaced time points and compared to the corresponding minimum-jerk reference using root-mean-square deviation:

$$D\_MJ - lat = \sqrt{}(1/N \cdot \Sigma\,(x\_i - x\_MJ, i)^2)$$

We report per-subject median and mean lateral deviations across the last five trials of left- and right-biased blocks for each cluster (Fig. S1). To further illustrate this metric, Figure S2 shows example observed trajectories together with their corresponding lateral minimum-jerk reference trajectories of all clusters.

Figure S1. Deviations from Lateral Minimum-Jerk-Trajectories

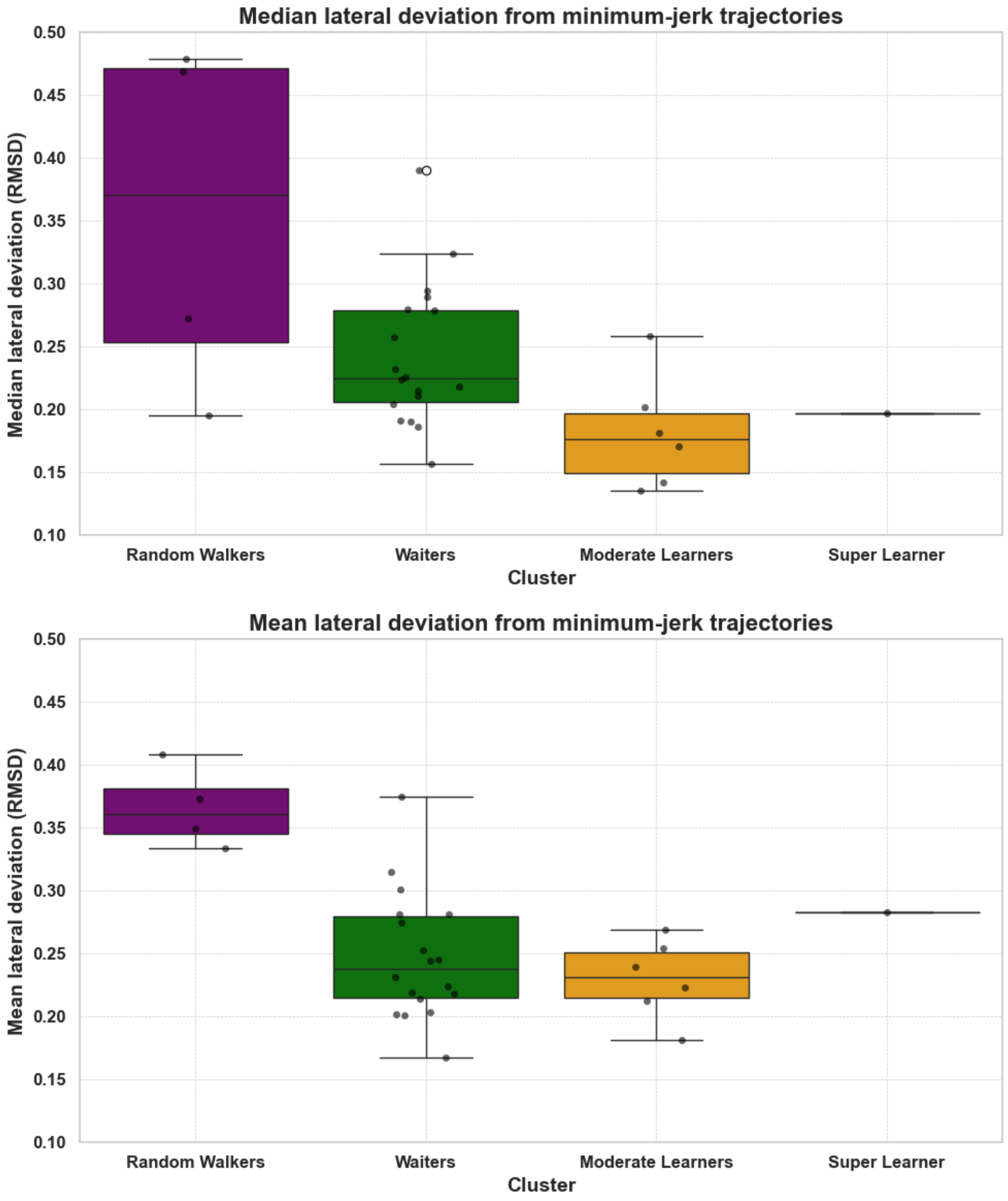

Figure S1. Boxplots (with individual subject points) show how strongly participants' observed walking trajectories in last 5 trials of left-biased and right-biased blocks deviated from smooth lateral minimum-jerk trajectories, quantified as RMSD of lateral displacement. Shown separately for the median (above) and mean (below) deviation per subject and cluster. The median provides a measure of typical deviation, whereas the mean is sensitive to occasional large corrections arising from an early commitment to the wrong side.

Figure S2. Representative Real and Minimum-Jerk Walking Trajectories for Each Cluster

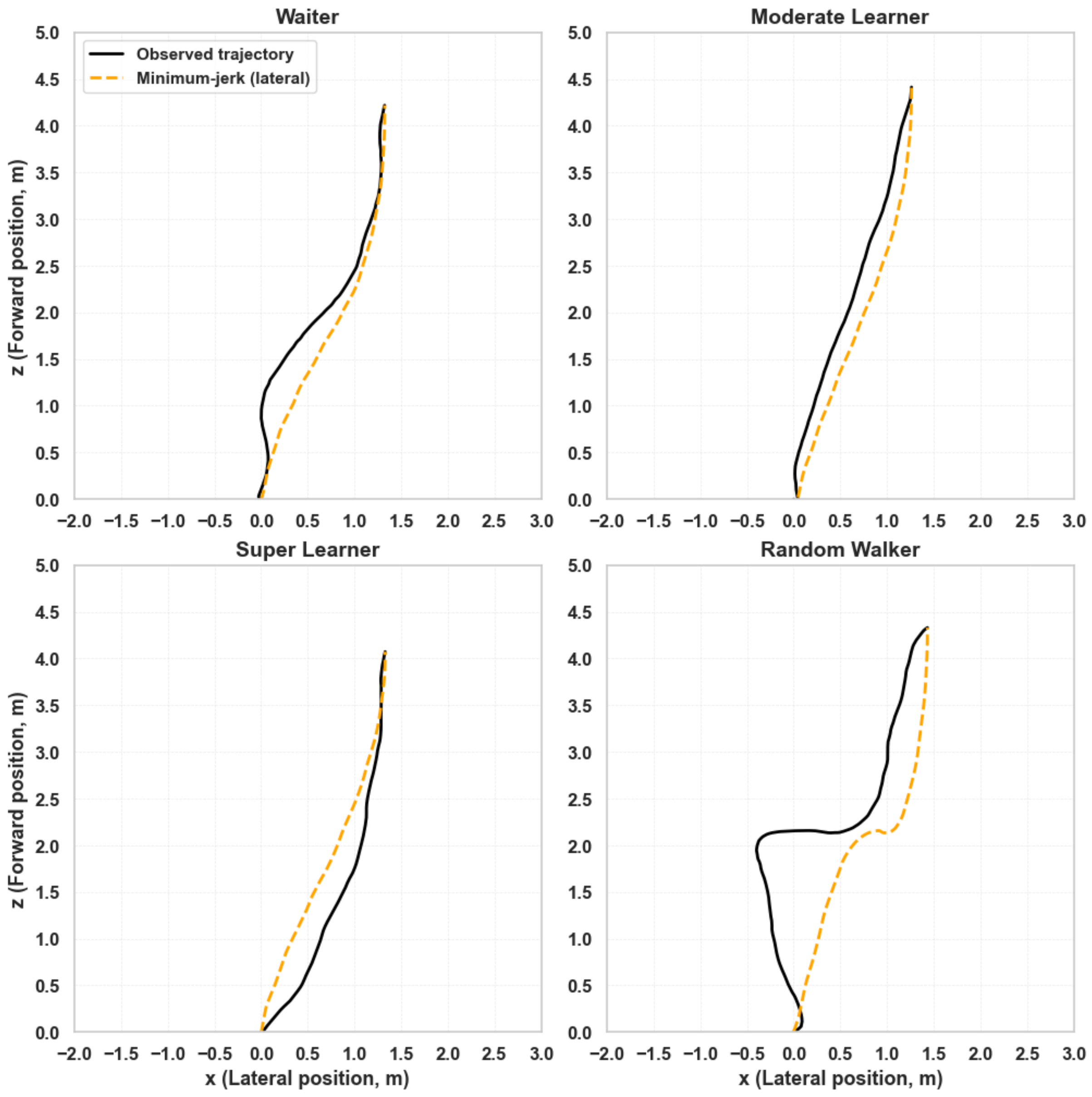

Figure S2. Representative examples of walking trajectories are shown together with their corresponding one-dimensional lateral minimum-jerk reference trajectories (dashed lines) for each cluster. Trajectories are plotted in participant-centered coordinates with forward displacement on the vertical axis and lateral displacement on the horizontal axis.

1.7. Gaze behavior

Table S6. Mixed-Effects Model: Saccade Rate

Fixed effects

Predictor	β	SE	z/t	p	95% CI low	95% CI high	Std. β
Intercept	1.03	0.103	9.967	<.001	0.828	1.233	—
Condition (control)	0.17	0.050	3.388	.001	0.071	0.267	0.093
Trial number	-0.008	0.004	-1.858	.063	-0.017	0.000	-0.054
Trial × Condition	-0.004	0.004	-1.021	.307	-0.013	0.004	-0.030
Block number	-0.052	0.009	-5.693	<.001	-0.070	-0.034	-0.156
Block × Trial	0.001	0.001	1.632	.103	-0.000	0.003	0.060

Random effects

Effect	Variance	SD	Corr. w/ intercept
Participant (Intercept)	0.234	0.484	—

Model fit

Marginal R^2 = 0.018
Conditional R^2 = 0.333

Table S7. Mixed-Effects Model: Fixation Dispersion

Fixed effects

Predictor	β	SE	z/t	p	95% CI low	95% CI high	Std. β
Intercept	0.549	0.046	12.029	<.001	0.459	0.638	—
Condition (control)	0.024	0.029	0.813	.416	-0.033	0.081	0.024
Trial number	-0.011	0.003	-4.364	<.001	-0.016	-0.006	-0.140
Trial × Condition	0.000	0.002	0.001	.999	-0.005	0.005	0.000
Block number	-0.041	0.005	-7.619	<.001	-0.052	-0.030	-0.229
Block × Trial	0.001	0.000	3.287	.001	0.001	0.002	0.133

Random effects

Effect	Variance	SD	Corr. w/ intercept
Participant (Intercept)	0.034	0.184	—

Model fit

Marginal R^2 = 0.025
Conditional R^2 = 0.189

2. Experiment 2

2.1 Trial-by-Trial Learning Models

Table S8. Logistic Mixed-Effects Model

Fixed effects

Predictor	β	SE	z/t	p	95% CI low	95% CI high	Odds Ratio	Std. β
Intercept	1.451	0.169	8.574	<.001	1.119	1.783	4.267	—
Trial number (z_trial)	0.450	0.049	9.120	<.001	0.353	0.547	1.568	0.450

Random effects

Effect	Variance	SD
Participant (Intercept)	0.650	0.806

Model fit

Marginal R^2 = 0.049
Conditional R^2 = 0.206
AIC = 2810.66

Table S9. Nonlinear Mixed-Effects Model

Fixed effects

Parameter	Estimate	SE	t	p	95% CI low	95% CI high
Asym	0.306	0.041	7.387	<.001	—	—
rate	0.031	0.005	6.623	<.001	—	—

Random effects

Effect	Variance	SD
Asym (random intercept)	0.037	0.192
Residual	0.147	0.383

Model fit

AIC = 2841.49
Log-Likelihood = -1416.74

2.2. Performance in Control Condition

Table S10. Logistic Mixed-Effects Model: Control Condition Pathway Choice

Fixed effects

Predictor	β	SE	z/t	p	95% CI low	95% CI high	Odds Ratio	Std. β
Intercept	0.052	0.063	0.823	.411	-0.072	0.176	1.053	—
Trial number (z_trial)	-0.087	0.063	-1.365	.172	-0.211	0.038	0.917	-0.087

Random effects

Effect	Variance	SD
Participant (Intercept)	0.000	0.000

Model fit

Marginal R^2 = 0.002
Conditional R^2 = 0.000

Figure S3. Correct Choices in Last 5 Trials of Control Blocks

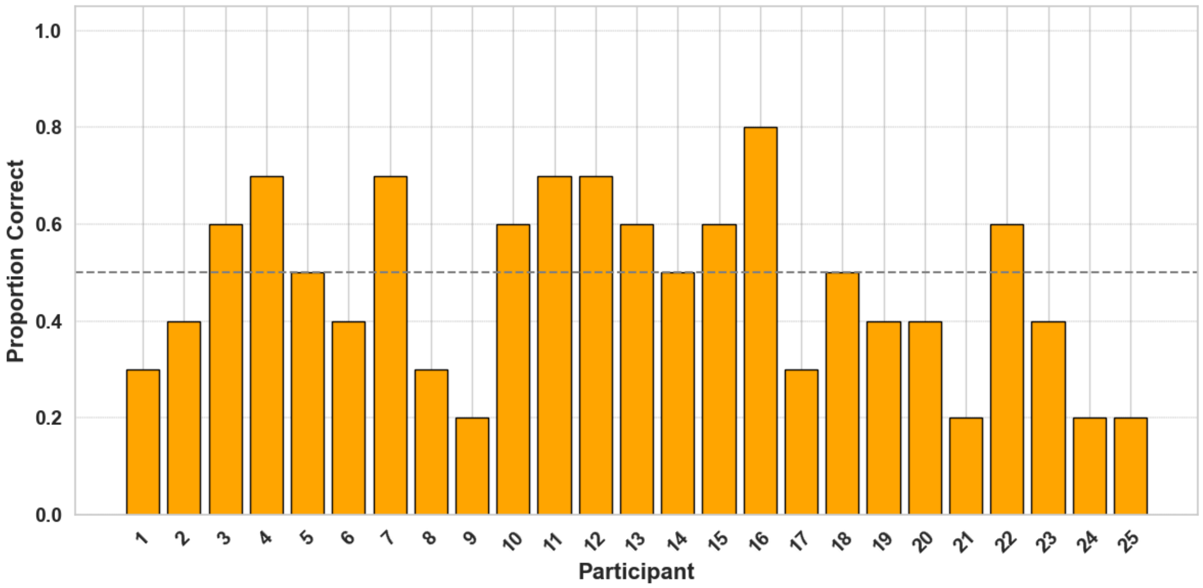

Figure S3. Proportion of correct pathway choices in the last five trials of each control block for all participants in Experiment 2 (averaged across control blocks). Bars show individual subject means; the dashed line marks chance level (0.5). As expected, performance in control blocks was highly variable and clustered around chance, indicating that no systematic lateral preferences or learning effects emerged in the absence of spatial regularities.

2.3. Learning Across Blocks

Table S11. Logistic Mixed-Effects Model

Fixed effects

Predictor	β	SE	z/t	p	95% CI low	95% CI high	Odds Ratio	Std. β
Intercept	1.464	0.171	8.565	<.001	1.129	1.799	4.324	—
Trial number (z_trial)	0.448	0.050	9.002	<.001	0.351	0.546	1.566	0.448
Block position (z_block)	0.217	0.049	4.394	<.001	0.120	0.314	1.243	0.217
z_trial × z_block	-0.055	0.049	-1.105	.269	-0.151	0.042	0.947	—

Random effects

Effect	Variance	SD
Participant (Intercept)	0.663	0.815

Model fit

Marginal R^2 = 0.060
Conditional R^2 = 0.218

2.4. Gaze behavior

Table S12. Mixed-Effects Model: Saccade Rate

Fixed effects

Predictor	β	SE	z/t	p	95% CI low	95% CI high	Std. β
Intercept	0.458	0.047	9.766	<.001	0.366	0.549	—
Condition (control)	0.025	0.038	0.652	.515	-0.050	0.099	0.026
Trial number	-0.005	0.003	-1.498	.134	-0.011	0.001	-0.064
Trial × Condition	-0.001	0.003	-0.294	.769	-0.007	0.005	-0.012
Block number	-0.041	0.007	-5.698	<.001	-0.056	-0.027	-0.228
Block × Trial	0.001	0.001	1.650	.099	-0.000	0.002	0.089

Random effects

Effect	Variance	SD
Participant (Intercept)	0.024	0.155

Model fit

Marginal R^2 = 0.030
Conditional R^2 = 0.168

Table S13. Mixed-Effects Model: Fixation Dispersion

Fixed effects

Predictor	β	SE	z/t	p	95% CI low	95% CI high	Std. β
Intercept	0.851	0.076	11.157	<.001	0.702	1.001	—
Condition (control)	0.021	0.060	0.357	.721	-0.097	0.140	0.014
Trial number	-0.018	0.005	-3.740	<.001	-0.027	-0.009	-0.157
Trial × Condition	0.002	0.005	0.401	.688	-0.008	0.012	0.017
Block number	-0.082	0.012	-7.152	<.001	-0.105	-0.060	-0.280
Block × Trial	0.002	0.001	2.431	.015	0.000	0.004	0.128

Random effects

Effect	Variance	SD
Participant (Intercept)	0.067	0.259

Model fit

Marginal R^2 = 0.046
Conditional R^2 = 0.196

2.5. Clustering in Experiment 2

To validate whether the clustering framework used in Experiment 1 generalizes to Experiment 2, we conducted two complementary analyses. Importantly, Experiment 2 differs fundamentally in task structure: participants must immediately commit to one of two spatially separated corridors, meaning that absolute movement offset is not behaviorally informative because the lateral displacement is imposed by the environment rather than chosen, and movement toward the correct direction is effectively equivalent to accuracy, as there is no opportunity for graded anticipatory adjustments. Thus, accuracy is the only meaningful behavioral indicator of learning in this experiment.

Accuracy-based clustering (1D analysis)

We computed subject-level accuracy in the last five trials of each left/right-biased block and averaged across blocks. A k-means clustering with $k = 2$ applied to this 1-dimensional measure produced a clean separation between the two Random Walkers and the remaining 23 Learners (Figure S2).

Figure S4. Accuracy-Based 1D Clustering

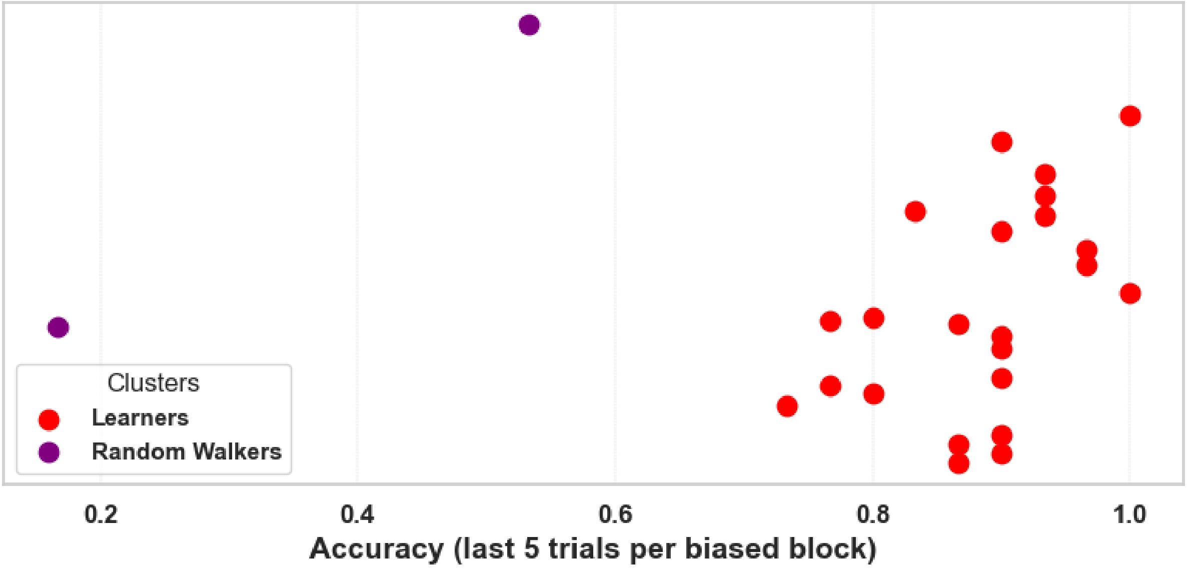

Figure S4. 1D k-means clustering (k = 2) based on subject-level accuracy in the last five trials of each biased block. Red = Learners; purple = Random Walkers.

Movement-based clustering (2D analysis)

For completeness and comparability with Experiment 1, we additionally repeated the movement-based clustering using absolute movement offset and movement toward the correct direction (weighted by $w = 2$). As expected, given the task structure, these metrics did not capture additional behavioral information beyond accuracy. Nevertheless, the 2D k-means solution with $k = 2$ reproduced the exact same separation as the 1D analysis, again isolating the two Random Walkers from the remaining Learners (Figure S3).

Figure S5. Movement-Based 2D Clustering

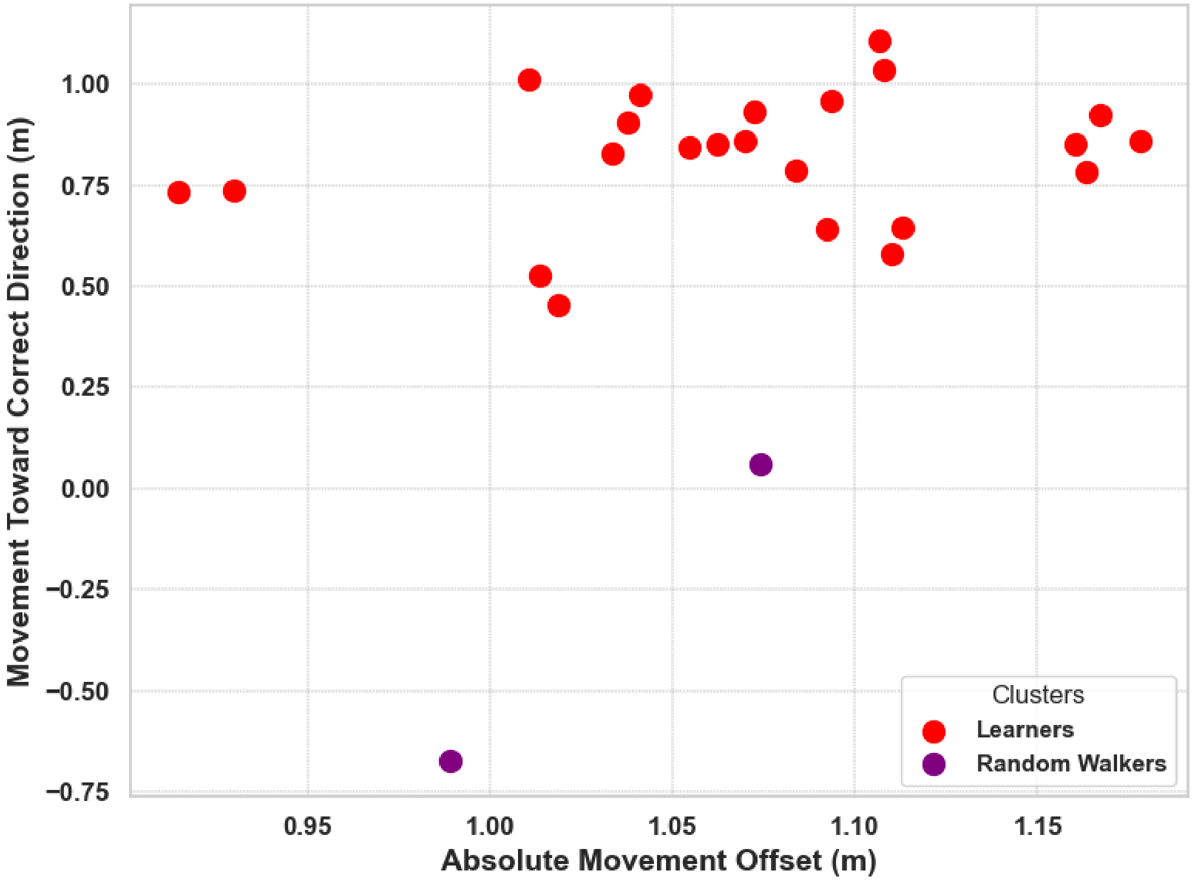

Figure S5. 2D k-means clustering (k = 2) using absolute movement offset and weighted directional movement. Red = Learners; purple = Random Walkers.

Figure S6. Representative Movement Trajectories of Learners and Random Walkers

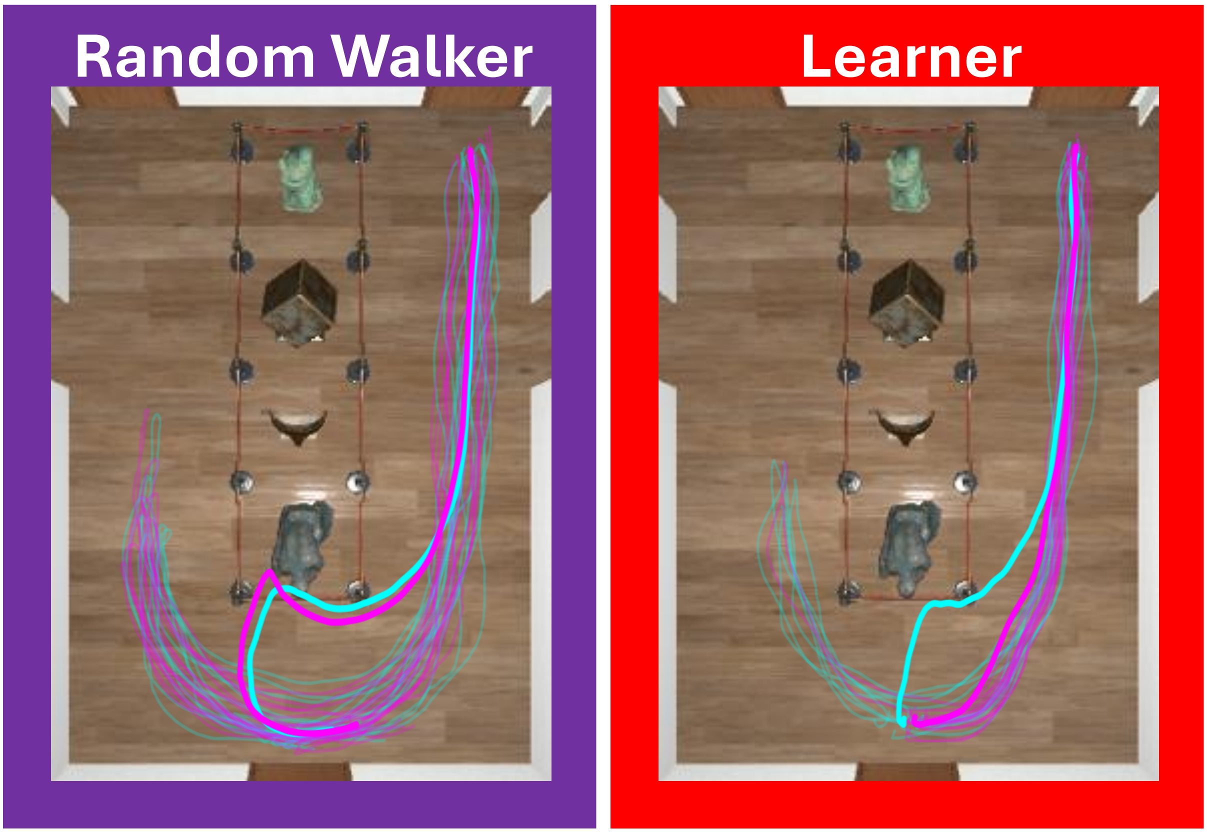

Figure S6. Example trajectory plots from one representative participant per cluster in Experiment 2, illustrating typical movement patterns in the first five and last five trials of *right-biased blocks* (i.e., blocks where the guard appeared on the left side). Thin blue lines represent individual trial trajectories from the first five trials; the bold blue line indicates their average. Thin pink lines represent individual trajectories from the last five trials; the thick pink line shows their average. For the Random Walker, characteristic return movements remain visible even in late trials, reflecting persistent incorrect initial choices and a lack of learning of the block-wise regularity.

References

1. Flash, T. & Hogan, N. The coordination of arm movements: an experimentally confirmed mathematical model. *J. Neurosci.* **5**, 1688–1703 (1985).
